# Enhancing Third- and Fifth-Order Nonlinearity via Tunneling in Multiple Quantum Dots

**DOI:** 10.3390/nano9030423

**Published:** 2019-03-12

**Authors:** Si-Cong Tian, Huan-Yu Lu, Hang Zhang, Li-Jie Wang, Shi-Li Shu, Xin Zhang, Guan-Yu Hou, Zi-Ye Wang, Cun-Zhu Tong, Li-Jun Wang

**Affiliations:** 1 State Key Laboratory of Luminescence and Applications, Changchun Institute of Optics, Fine Mechanics and Physics, Chinese Academy of Sciences, Changchun 130033, China; tiansicong@ciomp.ac.cn (S.-C.T.); 18744019056@163.com (H.-Y.L.); wanglijie@ciomp.ac.cn (L.-J.W.); shushili@ciomp.ac.cn (S.-L.S.); zhang315xin@ciomp.ac.cn (X.Z.); jumjim88@126.com (G.-Y.H.); wzywzy9625@foxmail.com (Z.-Y.W.); wanglj@ciomp.ac.cn (L.-J.W.); 2 University of Chinese Academy of Sciences, Beijing 100049, China; 3 Space Optics Department, Changchun Institute of Optics, Fine Mechanics and Physics, Chinese Academy of Sciences, Changchun 130033, China; zhanghang_ciomp@sina.com

**Keywords:** quantum dots, self-Kerr nonlinearity, fifth-order nonlinearity, cross-Kerr nonlinearity

## Abstract

The nonlinearity of semiconductor quantum dots under the condition of low light levels has many important applications. In this study, linear absorption, self-Kerr nonlinearity, fifth-order nonlinearity and cross-Kerr nonlinearity of multiple quantum dots, which are coupled by multiple tunneling, are investigated by using the probability amplitude method. It is found that the linear and nonlinear properties of multiple quantum dots can be modified by the tunneling intensity and energy splitting of the system. Most importantly, it is possible to realize enhanced self-Kerr nonlinearity, fifth-order nonlinearity and cross-Kerr nonlinearity with low linear absorption by choosing suitable parameters for the multiple quantum dots. These results have many potential applications in nonlinear optics and quantum information devices using semiconductor quantum dots.

## 1. Introduction

Nonlinear optical interaction of semiconductor quantum dots (QDs) has been widely studied [1,2,3,4,5,6,7,8,9,10] because it plays a fundamental role in many key devices, such as quantum logic gates [11,12], optical amplifiers [13,14] and single photon source [15]. One of the important goals of this field is to obtain large nonlinear interactions at low light levels. Electromagnetically-induced transparency (EIT), which is based on laser-induced atomic coherence, plays an important role in the interaction between light and matter [16,17,18]. With the help of EIT, Kerr nonlinearity can be greatly enhanced, and at the same time, the linear absorption can be suppressed [19,20,21,22,23,24,25,26,27], leading to the study of nonlinear optics at low light levels in EIT systems [28,29,30]. In addition, fifth-order nonlinearity has been studied in various mediums [31,32,33,34,35,36,37]. Fifth-order nonlinearity can have broad impacts in many fields, such as phase gate [38], multi-wave mixing [34,39,40] and optical solitons [35,41].

Two or more closely-spaced QDs can form quantum dot molecules (QDMs), in which the tunneling between the closely spaced QDs can induce atomic coherence and quantum interference. Vertical and lateral QDMs have been experimentally fabricated, and the number of QDs per molecule can be controlled via different growth conditions [42,43,44,45,46,47,48,49]. Many theoretical and experimental works on QDMs have been carried out [50,51,52,53,54,55,56,57,58,59,60,61,62,63,64,65]. In double quantum dots (DQDs), a phenomenon, which is similar to EIT and called tunneling induced transparency (TIT), has been reported [52]. Via TIT, giant self-Kerr nonlinearity with vanishing absorption can be realized in triple quantum dots (TQDs) [62].

Most of the studies of nonlinearity in QDMs are focused on self-Kerr nonlinearity. However, cross-Kerr nonlinearity and fifth-order nonlinearity can also be beneficial to many applications. In addition, most of the studies of nonlinearity are carried out in DQDs or TQDs. The study of nonlinearity in multiple quantum dots (MQDs) will bring extra controlling parameters and many novel results. It is also important to understand the mechanisms of other types of nonlinearities in MQDs and gain the ability to control them. In this study, the general analytic expression of linear and nonlinear susceptibility of the probe field in MQDs is deduced, and the linear absorption, self-Kerr nonlinearity, fifth-order nonlinearity and cross-Kerr nonlinearity of MQDs is then investigated. The linear and nonlinear properties of MQDs can be modified by the parameters of MQDs, and it is possible to realize enhanced self-Kerr nonlinearity, fifth-order nonlinearity and cross-Kerr nonlinearity with low linear absorption.

## 2. Models and Equations

**Figure 1:**
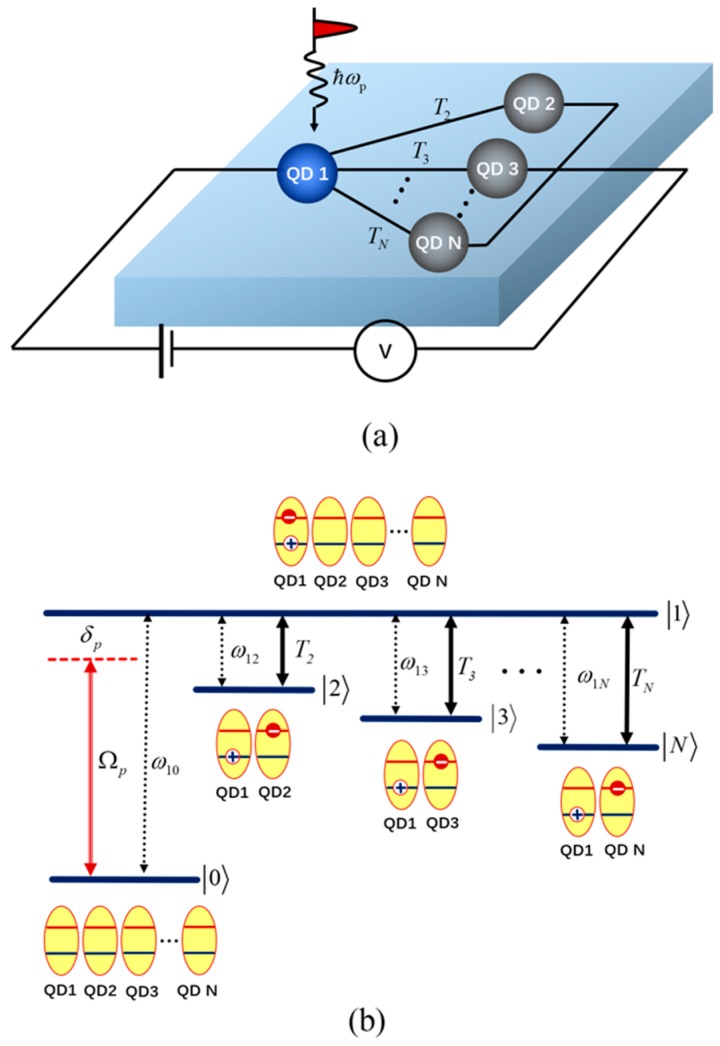
(**a**) The schematic of the setup of a multiple quantum dots (MQDs); (**b**) the schematic of the level configuration of a MQDs.

A schematic of the setup of the MQDs is shown in Figure 1a. The number of QDs is *N* in MQDs. *QD1* and *QDn* (*n* = 2, 3,…*N*) are coupled by gate electrodes, and there is no gate electrode between *QDn* (*n* = 2,3,…*N*). When no gate voltage between *QD1* and *QDn* is applied, the electron tunneling between *QD1* and *QDn* is very weak because the conduction-band electron levels are not resonant. While the gate voltage is applied, the electron tunneling between *QD1* and *QDn* is greatly increased because the conduction-band electron levels come close to resonance. The hole tunneling is neglected because of the far off-resonant valence-band energy levels. A schematic of the level configuration of the MQDs is shown in Figure 1b. |0〉 is the ground state without excitations, |1〉 is the exciton state with one electron-hole pair being in QD1, and |n〉 (n=2,3,…N) is the indirect exciton state with the electron being in *nth* QD and the hole remaining in QD1.

A weak probe field with a Rabi frequency of $\Omega_{p}=\mu_{01}E_{p}$ and detuning of $\delta_{p}=\omega_{10}-\omega_{p}$ probes the transition of |0〉→|1〉, with $\mu_{01}$ being the electric dipole moment for the excitonic transition between states |0〉 and |1〉, $E_{p}$ being the electric-field amplitude of the probe field, $\omega_{10}$ being the energy splitting between states |0〉 and s |1〉, and $\omega_{p}$ being the frequency of the probe field. The *nth* tunneling couples the transition from state |n〉 (n=2,3,…N) to state |1〉. The intensity of the *nth* tunneling is $T_{n}\;(n=2,3,\ldots N)$, depending on the barrier characteristics and the external electric field. $\omega_{1n}\;(n=2,3,\ldots N)$ is the energy splitting between the exciton state |1〉 and indirect exciton state |n〉 (n=2,3,…N), and can be controlled by manipulation of the external electric field which changes the effective confinement potential.

The Hamiltonian of the basis {|0〉,|1〉,…|N〉} under the rotating-wave and the electric-dipole approximations can be written as (assumption of ℏ=1):

(1) $$H_{I}=\left(\begin{array}{ccccc} 0 & -\Omega_{p} & 0 & \ldots & 0\\ -\Omega_{p} & \delta_{p} & -T_{2} & \ldots & -T_{N}\\ 0 & -T_{2} & \delta_{p}-\omega_{12} & \ldots & 0\\ \ldots & \ldots & \ldots & \ldots & \ldots \\ 0 & -T_{N} & 0 & \ldots & \delta_{p}-\omega_{1N} \end{array}\right)$$

The state vector at any time *t* is:

(2) $$|\Psi_{I}(t)\rangle =\sum_{n=0}^{N}a_{n}(t)|n\rangle$$

$a_{n}(t)$ is the atomic probability amplitude of state |n〉. The evolution of the state vector obeys the Schrödinger equation:

(3) $$\frac{d}{dt}|\Psi_{I}(t)\rangle =-iH_{I}(t)|\Psi_{I}(t)\rangle$$

Substituting Equations (1) and (2) into Equation (3), and then using the Weisskopf–Wigner theory can obtain the dynamical equations for atomic probability amplitudes in the interaction picture:

(4) $$i{\dot{a}}_{0}=-\Omega_{p}a_{1},$$

(5) $$i{\dot{a}}_{1}=-\Omega_{p}a_{0}-\sum_{n=2}^{N}T_{n}a_{n}+\left(\delta_{p}-i\gamma_{1}\right)a_{1},$$

(6) $$i{\dot{a}}_{n}=-T_{n}a_{1}+\left(\delta_{p}-\omega_{1n}-i\gamma_{n}\right)a_{n}\;(n=2,3,\ldots ,N),$$

(7) $${\sum_{n=0}^{N}\left|a_{n}\right|}^{2}=1,$$

where $\gamma_{n}=\Gamma_{n0}/2+\gamma_{n0}^{d}\;(n=1,2,\ldots N)$ is the typical effective decay rate, $\Gamma_{n0}$ is the radiative decay rate of populations from states |n〉→|0〉 and $\gamma_{n0}^{d}$ is the pure dephasing rate. The response of MQDs to the probe field is governed by susceptibility $\chi_{p}=\frac{\Gamma }{V}\frac{\mu_{01}^{2}}{\varepsilon_{0}\hbar \Omega_{p}}\chi$. Γ is the optical confinement factor, V is the volume of MQDs, $\varepsilon_{0}$ is the dielectric constant, and $\mu_{01}$ is the associated dipole transition-matrix element.

By solving Equations (4)–(7) under the weak field approximation, the analytical expression of χ can be obtained (Appendix A):

(8) $$\chi =\frac{1}{\Gamma_{1}-\sum_{n=2}^{N}\frac{T_{n}^{2}}{\Gamma_{n}}}\cdot \frac{1}{1+\frac{\Omega_{p}^{2}}{{\left|\Gamma_{1}-\sum_{n=2}^{N}\frac{T_{n}^{2}}{\Gamma_{n}}\right|}^{2}}\left(1+\sum_{n=2}^{N}\frac{T_{n}^{2}}{{\left|\Gamma_{n}\right|}^{2}}\right)},$$

where $\Gamma_{1}=\delta_{p}-i\gamma_{1}$ and $\Gamma_{n}=\delta_{p}-\omega_{1n}-i\gamma_{n}\;(n=2,3,\ldots ,N)$.

The linear susceptibility is proportional to $\Omega_{p}^{0}$, the third-order susceptibility is proportional to $\Omega_{p}^{2}$ and the fifth-order susceptibility is proportional to $\Omega_{p}^{4}$. By using Maclaurin formula, χ can be expended into the fourth-order of $\Omega_{p}$,

(9) $$\chi =\chi^{\left(1\right)}+\chi^{\left(3\right)}\Omega_{p}^{2}+\chi^{\left(5\right)}\Omega_{p}^{4},$$

where

(10) $$\chi^{\left(1\right)}=-\frac{1}{\Gamma_{1}-\sum_{n=2}^{N}\frac{T_{n}^{2}}{\Gamma_{n}}},$$

(11) $$\chi^{\left(3\right)}=-\frac{1}{\Gamma_{1}-\sum_{n=2}^{N}\frac{T_{n}^{2}}{\Gamma_{n}}}\cdot \frac{1}{{\left|\Gamma_{1}-\sum_{n=2}^{N}\frac{T_{n}^{2}}{\Gamma_{n}}\right|}^{2}}\cdot \left(1+\sum_{n=2}^{N}\frac{T_{n}^{2}}{{\left|\Gamma_{n}\right|}^{2}}\right),$$

(12) $$\chi^{\left(5\right)}=-\frac{1}{\Gamma_{1}-\sum_{n=2}^{N}\frac{T_{n}^{2}}{\Gamma_{n}}}\cdot \frac{1}{{\left|\Gamma_{1}-\sum_{n=2}^{N}\frac{T_{n}^{2}}{\Gamma_{n}}\right|}^{4}}\cdot {\left(1+\sum_{n=2}^{N}\frac{T_{n}^{2}}{{\left|\Gamma_{n}\right|}^{2}}\right)}^{2}.$$

Then by using the method in References [26,27], the cross-Kerr nonlinearity (cross phase modulation) effect induced by tunneling $T_{i}$ ($\Omega_{p}<T_{i}<T_{n}\;(n\neq i)$) on the probe field can be given as:

(13) $$\chi_{Ti}^{\left(1\right)}=-\frac{1}{\Gamma_{1}-\sum_{n=2,n\neq i}^{N}\frac{T_{n}^{2}}{\Gamma_{n}}},$$

(14) $$\chi_{Ti}^{\left(3\right)}=-\frac{1}{\Gamma_{i}{\left(\Gamma_{1}-\sum_{n=2,n\neq i}^{N}\frac{T_{n}^{2}}{\Gamma_{n}}\right)}^{2}}.$$

The linear absorption Im[χ(1)] corresponds to the imaginary part of the first-order susceptibility, the self-Kerr nonlinearity Re[χ(3)] corresponds to the real part of the third-order susceptibility, the fifth-order nonlinearity Re[χ(5)] corresponds to the real part of the fifth-order susceptibility, and the cross-Kerr nonlinearity Re[χTi(3)] corresponds to the real part of the third-order susceptibility $\chi_{Ti}^{\left(3\right)}$ between probe field and tunneling $T_{i}$.

## 3. Results and Discussion

The number of QDs in MQDs is five in the following calculation and discussion. Therefore, the intensity of tunneling are $T_{2}$, $T_{3}$, $T_{4}$ and $T_{5}$, respectively. The energy splittings are $\omega_{12}$, $\omega_{13}$, $\omega_{14}$ and $\omega_{15}$, respectively. The typical effective decay rate for each state are $\gamma_{1}$, $\gamma_{2}$, $\gamma_{3}$, $\gamma_{4}$ and $\gamma_{5}$, respectively. In this study, all parameters are scaled by the decay rate $\gamma_{1}$.

In MQDs, the tunneling couplings $T_{i}$ depends on the barrier characteristics and the external electric field, frequency transition $\omega_{1i}$ depends on effective confinement potential which can be manipulated by the external electric field. MQDs have been achieved in much experimental progress [42,43,44,45,46,47,48,49], and the realistic values of the parameters are according to References [52,62] and references therein. In addition, the tunneling can be in weak [66] or strong tunneling regime [67]. Some of the value of parameters are for DQDs or TQDs, however, it can be inferred that the tunneling, frequency transition and decay rates of MQDs have the same order as those of DQDs or TQDs.

Based on the above equations, the linear absorption Im[χ(1)], self-Kerr nonlinearity Re[χ(3)], fifth-order nonlinearity Re[χ(5)] and cross-Kerr nonlinearity Re[χTi(3)] between the probe field and tunneling $T_{i}$ are investigated for varying of tunneling intensity and energy splitting.

### 3.1. Tunneling Induced Transparency of MQDs

**Figure 2:**
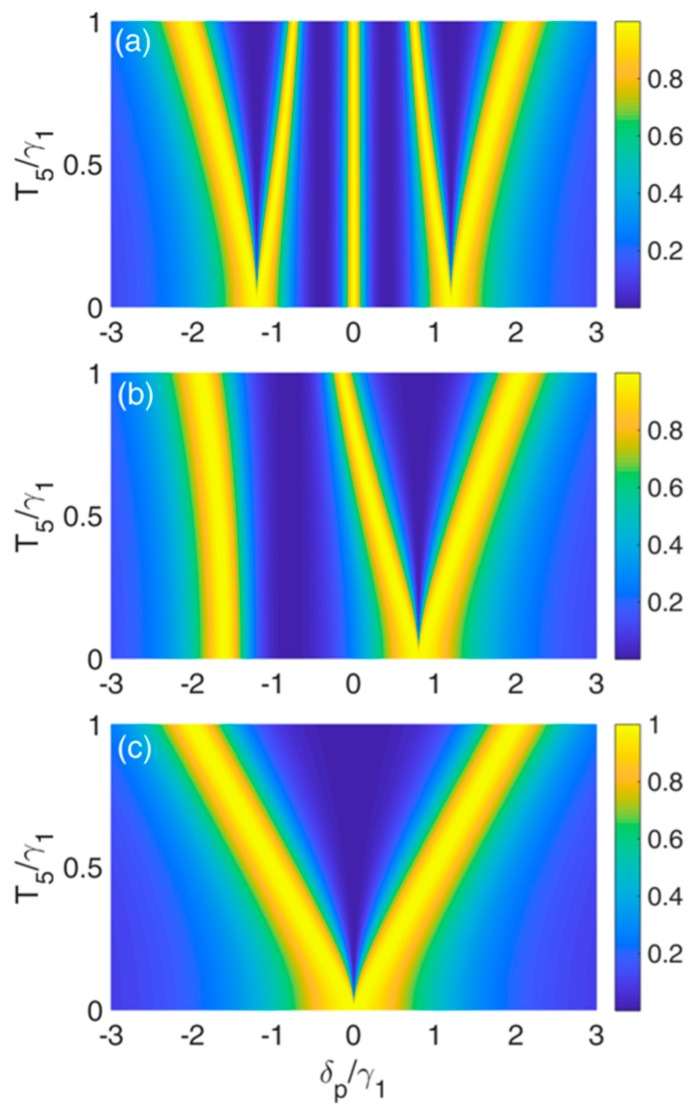
Linear absorption Im[χ(1)] as functions of probe detuning $\delta_{p}$ and tunneling intensity $T_{5}$ for varying conditions of the energy splitting. (**a**) $\omega_{12}=-1.2\gamma_{1}$, $\omega_{13}=-0.4\gamma_{1}$, $\omega_{14}=0.4\gamma_{1}$, $\omega_{15}=1.2\gamma_{1}$, $T_{3}=T_{4}=0.8\gamma_{1}$, $T_{2}=T_{5}$; (**b**) $\omega_{12}=\omega_{13}=-0.8\gamma_{1}$, $\omega_{14}=\omega_{15}=0.8\gamma_{1}$, $T_{2}=T_{3}=0.8\gamma_{1}$, $T_{4}=T_{5}$; (**c**) $\omega_{12}=\omega_{13}=\omega_{14}=\omega_{15}=0$, $T_{2}=T_{3}=T_{4}=T_{5}$. The other parameters are $\gamma_{2}=\gamma_{3}=\gamma_{4}=\gamma_{5}=10^{-3}\gamma_{1}$.

In Figure 2, the 2D linear absorption Im[χ(1)] as functions of probe detuning $\delta_{p}$ and tunneling intensity $T_{5}$ for varying energy splitting are investigated. Firstly, when all values of energy splitting are not equal, that is $\omega_{12}\neq \omega_{13}\neq \omega_{14}\neq \omega_{15}$, it can be seen from Figure 2a that there are five absorption peaks and four TIT windows. By decreasing value of $T_{2}$ and $T_{5}$, the width of the outer side of TIT windows becomes much narrowing, however, these TIT windows always locate at $\delta_{p}=\omega_{12}$, $\delta_{p}=\omega_{13}$, $\delta_{p}=\omega_{14}$, and $\delta_{p}=\omega_{15}$.

Secondly, in the case of $\omega_{12}=\omega_{13}\neq \omega_{14}=\omega_{15}$, it can be seen from Figure 2b that there are only three absorption peaks and two TIT windows. By decreasing tunneling intensity $T_{4}$ and $T_{5}$ at the same time, the right-hand side of TIT window becomes much narrowing, while that of the left-hand side becomes a little widen. The two TIT windows locate at $\delta_{p}=\omega_{12}$ and $\delta_{p}=\omega_{14}$.

Thirdly, with $\omega_{12}=\omega_{13}=\omega_{14}=\omega_{15}$, only two absorption peaks and one TIT window is obtained as shown in Figure 2c. By decreasing all tunneling intensity, the width of the TIT window narrows, and the profile of Im[χ(1)] keeps symmetry.

In all three cases, Im[χ(1)] can be controlled by the tunneling intensity and energy splitting, especially, narrow TIT window can be obtained by choosing the suitable parameters, which is essential to acquire enhancement of nonlinearity. The TIT window also locates at $\delta_{p}=\omega_{12}$.

### 3.2. Dressed States of MQDs

**Figure 3:**
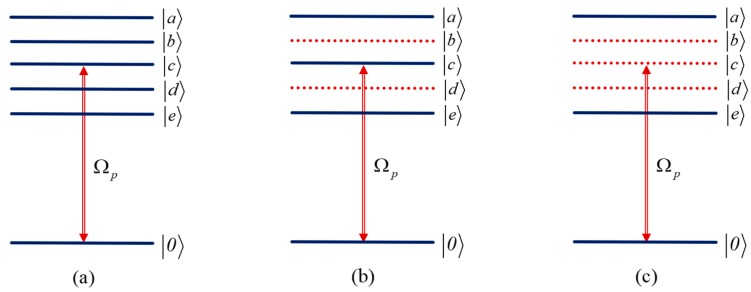
Dressed states of the MQDs with five dots under the tunneling coupling and for varying condition of energy splitting of the exciton states, (**a**) $\omega_{12}\neq \omega_{13}\neq \omega_{14}\neq \omega_{15}$; (**b**) $\omega_{12}=\omega_{13}\neq \omega_{14}=\omega_{15}$; (**c**) $\omega_{12}=\omega_{13}=\omega_{14}=\omega_{15}$.

The corresponding results in Figure 2 can be explained under the dressed state picture. Under the coupling of four tunneling fields, the system can be treated as a new system under the dressed states, as shown in Figure 3. Each dressed state is the combination of the bare states |1〉, |2〉, |3〉, |4〉 and |5〉. Therefore, the weak probe field probes the transition from ground state |0〉 to the dressed state |i〉 (i=a,b,c,d,e).

In the case of $\omega_{12}\neq \omega_{13}\neq \omega_{14}\neq \omega_{15}$, each dressed state has the component of state |1〉 (Figure 3a), resulting in five absorption peaks at five different detuning of the probe field. In addition, quantum interference between the transitions |0〉→|i〉 (i=a,b,c,d,e) will lead to four TIT windows (Figure 2a). By choosing the suitable tunneling intensity and energy splitting of the MQDs, the width of TIT windows can be very narrow. The narrowing of the TIT window is responsible for acquiring enhanced nonlinearity, which is the basis for the following calculation and discussion.

In the case of $\omega_{12}=\omega_{13}\neq \omega_{14}=\omega_{15}$, the dressed states |b〉 and |d〉 do not have the component of state |1〉 anymore (Figure 3b), so there are three absorption peaks and two TIT windows resulted from quantum interference between the transitions |0〉→|a〉, |0〉→|c〉 and |0〉→|e〉 (Figure 2b).

If all the energy splitting is equal, that is $\omega_{12}=\omega_{13}=\omega_{14}=\omega_{15}$, the dressed states |b〉, |c〉 and |d〉 do not have the component of state |1〉 anymore (Figure 3c), so there are only two absorption peaks and one TIT window resulted from quantum interference between the transitions |0〉→|a〉 and |0〉→|e〉 (Figure 2c). In all these cases, one can modify the width of the TIT windows via the tunneling coupling, as shown in Figure 2.

### 3.3. Self-Kerr and Fifth-Order Nonlinearity of MQDs

According to Equations (10) and (11), the linear absorption Im[χ(1)] and self-Kerr nonlinearity Re[χ(3)] are calculated for three different conditions of energy splitting.

**Figure 4:**
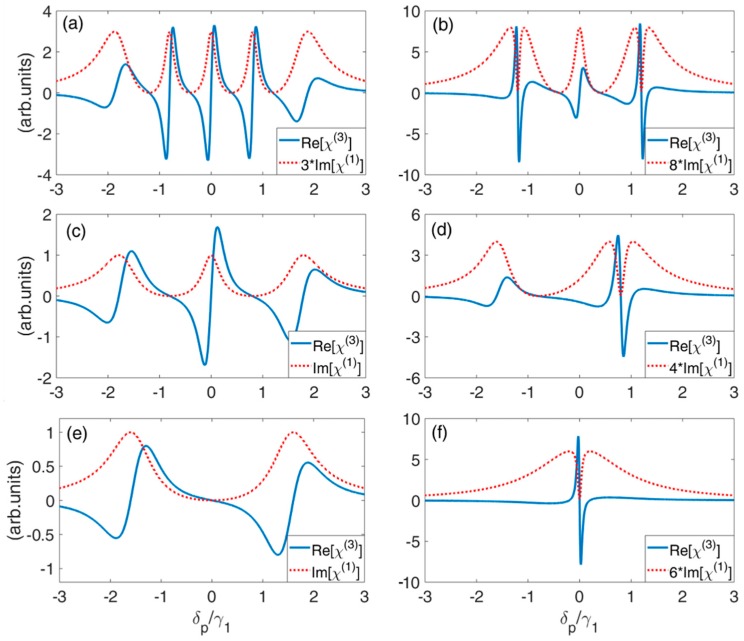
Linear absorption Im[χ(1)] and self-Kerr nonlinearity Re[χ(3)] as a function of probe detuning $\delta_{p}$ for varying tunneling coupling and energy splitting. (**a**) $\omega_{12}=-1.2\gamma_{1}$, $\omega_{13}=-0.4\gamma_{1}$, $\omega_{14}=0.4\gamma_{1}$, $\omega_{15}=1.2\gamma_{1}$, $T_{3}=T_{4}=0.8\gamma_{1}$, $T_{2}=T_{5}=0.8\gamma_{1}$; (**b**) the parameters are the same as those in (**a**), except $T_{2}=T_{5}=0.2\gamma_{1}$; (**c**) $\omega_{12}=\omega_{13}=-0.8\gamma_{1}$, $\omega_{14}=\omega_{15}=0.8\gamma_{1}$, $T_{2}=T_{3}=0.8\gamma_{1}$, $T_{4}=T_{5}=0.8\gamma_{1}$; (**d**) the parameters are the same as those in (**c**), except $T_{4}=T_{5}=0.2\gamma_{1}$; (**e**) $\omega_{12}=\omega_{13}=\omega_{14}=\omega_{15}=0$, $T_{2}=T_{3}=T_{4}=T_{5}=0.8\gamma_{1}$; (**f**) the parameters are the same as those in (**e**), except $T_{2}=T_{3}=T_{4}=T_{5}=0.1\gamma_{1}$. The other parameters are $\gamma_{2}=\gamma_{3}=\gamma_{4}=\gamma_{5}=10^{-3}\gamma_{1}$.

Firstly, in Figure 4a,b, Im[χ(1)] and Re[χ(3)] as a function of probe detuning $\delta_{p}$ under the condition of $\omega_{12}\neq \omega_{13}\neq \omega_{14}\neq \omega_{15}$ is investigated. Figure 4a shows that, with equal value of tunneling intensity, Im[χ(1)] curve exhibits symmetric properties with five absorption peaks and four TIT windows. In addition, Re[χ(3)] is enhanced with strong absorption, which is not suitable for applications. Then in Figure 4b, the energy splitting is kept unchanged, but tunneling intensity $T_{2}$ and $T_{5}$ is reduced. The width of the outer side of the TIT window becomes narrower, at the same time, in the vicinity of the TIT windows, enhanced self-Kerr nonlinearity is realized. In addition, compared with the results of TQDs [62], enhanced self-Kerr nonlinearity within the vicinity of TIT window can occur at two probe detuning, where $\delta_{p}=\omega_{12}$ and $\delta_{p}=\omega_{15}$.

Secondly, in Figure 4c,d, Im[χ(1)] and Re[χ(3)] as a function of probe detuning $\delta_{p}$ under the condition of $\omega_{12}=\omega_{13}=-0.8\gamma_{1}$ and $\omega_{14}=\omega_{15}=0.8\gamma_{1}$ is investigated. With equal value of tunneling intensity, Im[χ(1)] curve exhibits symmetric property with two TIT windows locating at $\delta_{p}=\pm 0.8\gamma_{1}$. This is similar like the one realized in TQDs. Re[χ(3)] is enhanced with large absorption in the vicinity of the absorption peaks, as shown in Figure 4c. Then with smaller intensity of tunneling $T_{4}$ and $T_{5}$, the right side of the TIT window becomes much narrower, and enhanced Re[χ(3)] enters the narrow TIT window, as shown in Figure 4d. This means that enhanced self-Kerr nonlinearity with low absorption can be obtained.

Thirdly, in Figure 4e,f, Im[χ(1)] and Re[χ(3)] as a function of probe detuning $\delta_{p}$ under the condition of $\omega_{12}=\omega_{13}=\omega_{14}=\omega_{15}=0$ is investigated. As can be seen that there is only one TIT window at $\delta_{p}=0$, however, self-Kerr nonlinearity is not enhanced (Figure 4e). Then with smaller value of all tunneling, the width of TIT window becomes narrower. In the vicinity of this TIT window, enhanced self-Kerr nonlinearity occurs (Figure 4f).

These results reveal that one can obtain enhanced self-Kerr nonlinearity by choosing the suitable parameters of tunneling intensity and energy splitting. Compared with QDMs with two or three dots [62], more than one probe fields with different frequencies can acquire enhanced Kerr nonlinearity simultaneously.

**Figure 5:**
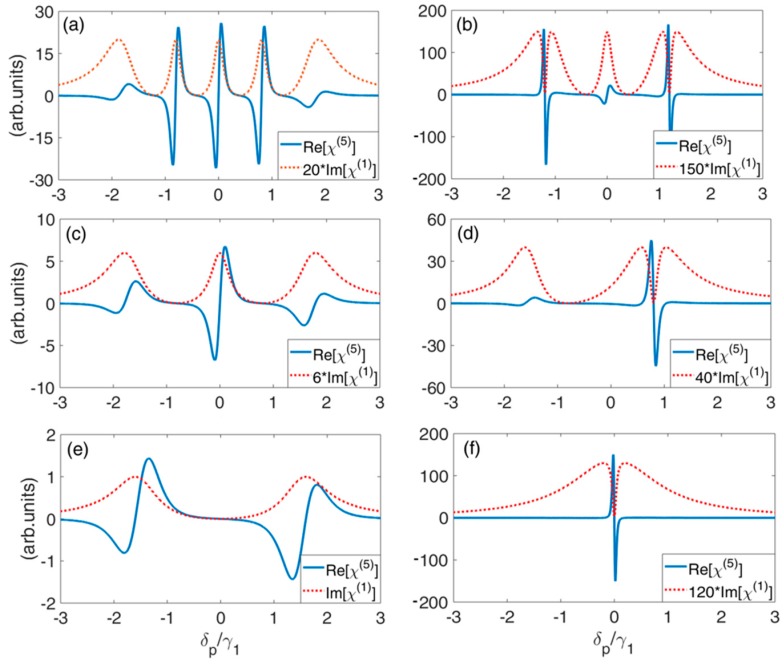
Linear absorption Im[χ(1)] and fifth-order nonlinearity Re[χ(5)] as a function of probe detuning $\delta_{p}$ for varying tunneling coupling and energy splitting. (**a**) $\omega_{12}=-1.2\gamma_{1}$, $\omega_{13}=-0.4\gamma_{1}$, $\omega_{14}=0.4\gamma_{1}$, $\omega_{15}=1.2\gamma_{1}$, $T_{3}=T_{4}=0.8\gamma_{1}$, $T_{2}=T_{5}=0.8\gamma_{1}$; (**b**) the parameters are the same as those in (**a**), except $T_{2}=T_{5}=0.2\gamma_{1}$; (**c**) $\omega_{12}=\omega_{13}=-0.8\gamma_{1}$, $\omega_{14}=\omega_{15}=0.8\gamma_{1}$, $T_{2}=T_{3}=0.8\gamma_{1}$, $T_{4}=T_{5}=0.8\gamma_{1}$; (**d**) the parameters are the same as those in (**c**), except $T_{4}=T_{5}=0.2\gamma_{1}$; (**e**) $\omega_{12}=\omega_{13}=\omega_{14}=\omega_{15}=0$, $T_{2}=T_{3}=T_{4}=T_{5}=0.8\gamma_{1}$; (**f**) the parameters are the same as those in (**e**), except $T_{2}=T_{3}=T_{4}=T_{5}=0.1\gamma_{1}$. The other parameters are $\gamma_{2}=\gamma_{3}=\gamma_{4}=\gamma_{5}=10^{-3}\gamma_{1}$.

For the susceptibility magnitude decreases typically with increasing order of nonlinearity, most nonlinear studies at low light level have focused on the third-order processes. So here, the fifth-order nonlinearity is also investigated. According to Equations (10) and (12), the linear absorption Im[χ(1)] and fifth-order nonlinearity Re[χ(5)] as a function of probe detuning $\delta_{p}$ for three different conditions of energy splitting are plotted in Figure 5. Using the same parameters in Figure 4, Re[χ(5)] exhibits the similar profile. Enhanced fifth-order nonlinearity can be obtained in the vicinity of TIT windows by choosing the suitable tunneling intensity and energy splitting. Compared with Reference [63], more than one probe fields with different frequencies can acquire enhanced fifth-order nonlinearity simultaneously.

### 3.4. Cross-Kerr Nonlinearity of MQDs

In this section, according to Equations (10) and (14), cross-Kerr nonlinearity Re[χTi(3)] between probe field and the *ith* tunneling field as a function of probe detuning $\delta_{p}$ under different condition of energy splitting is investigated.

**Figure 6:**
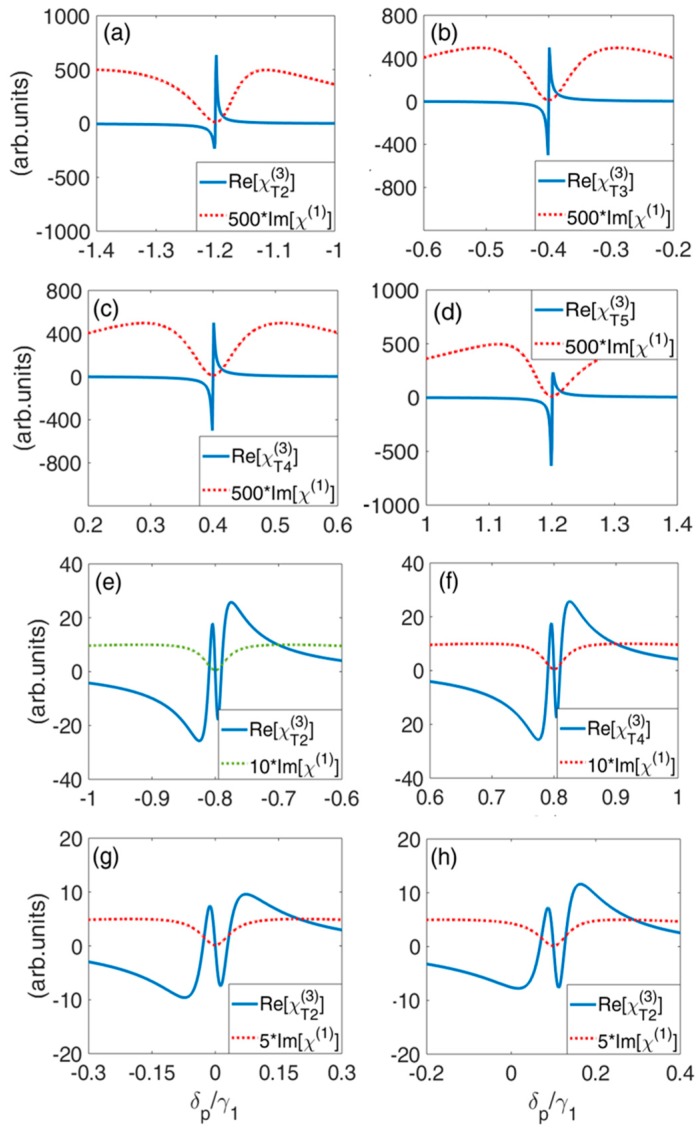
Linear absorption Im[χ(1)] and cross-Kerr nonlinearity Re[χTi(3)] as a function of probe detuning $\delta_{p}$ for varying tunneling coupling and energy splitting. (**a**) Re[χT2(3)], the parameters are $\omega_{12}=-1.2\gamma_{1}$, $\omega_{13}=-0.4\gamma_{1}$, $\omega_{14}=0.4\gamma_{1}$, $\omega_{15}=1.2\gamma_{1}$, $T_{3}=T_{4}=T_{5}=0.8\gamma_{1}$; (**b**) Re[χT3(3)], the parameters are the same as those in (**a**), except $T_{2}=T_{4}=T_{5}=0.8\gamma_{1}$; (**c**) Re[χT4(3)], the parameters are the same as those in (**a**), except $T_{2}=T_{3}=T_{5}=0.8\gamma_{1}$; (**d**) Re[χT5(3)], the parameters are the same as those in (**a**), except $T_{2}=T_{3}=T_{4}=0.8\gamma_{1}$; (**e**) Re[χT2(3)] (Re[χT3(3)]), $\omega_{12}=\omega_{13}=-0.8\gamma_{1}$, $\omega_{14}=\omega_{15}=0.8\gamma_{1}$, $T_{3}=0.1\gamma_{1}$, $T_{4}=T_{5}=0.8\gamma_{1}$; (**f**) Re[χT4(3)] (Re[χT5(3)]), the parameters are the same as those in (**e**), except $T_{2}=T_{3}=0.8\gamma_{1}$, $T_{5}=0.1\gamma_{1}$; (**g**) Re[χT2(3)] (Re[χT3(3)], Re[χT4(3)] and Re[χT5(3)]), $\omega_{12}=\omega_{13}=\omega_{14}=\omega_{15}=0$, $T_{3}=T_{4}=T_{5}=0.1\gamma_{1}$; (**h**) Re[χT2(3)] (Re[χT3(3)], Re[χT4(3)] and Re[χT5(3)]), $\omega_{12}=\omega_{13}=\omega_{14}=\omega_{15}=0.1\gamma_{1}$, $T_{3}=T_{4}=T_{5}=0.1\gamma_{1}$. The other parameters are $\gamma_{2}=\gamma_{3}=\gamma_{4}=\gamma_{5}=10^{-3}\gamma_{1}$.

Firstly, in Figure 6a–d, cross-Kerr nonlinearity between probe field and $T_{2}$, $T_{3}$, $T_{4}$ and $T_{5}$ is calculated, respectively. It can be seen that for each tunneling enhanced cross-Kerr nonlinearity can be realized with low linear absorption. The advantage of realizing cross-Kerr nonlinearity in MQDs system is that such enhanced cross-Kerr nonlinearity with low linear absorption can be achieved for probe fields with different frequencies.

Secondly, cross-Kerr nonlinearity Re[χTi(3)] between probe field and the *ith* tunneling field under the condition of $\omega_{12}=\omega_{13}$ and $\omega_{14}=\omega_{15}$ is shown in Figure 6e,f. The curves of Re[χT2(3)] and Re[χT3(3)] are coincident (Figure 6e), and that of Re[χT4(3)] and Re[χT5(3)] are also coincident (Figure 6f). It is shown that enhanced cross-Kerr nonlinearity with low linear absorption can also be realized.

Thirdly, cross-Kerr nonlinearity Re[χTi(3)] between probe field and the *ith* tunneling field under the condition of $\omega_{12}=\omega_{13}=\omega_{14}=\omega_{15}$ is shown in Figure 6g,f. The curves of Re[χT2(3)], Re[χT3(3)], Re[χT4(3)] and Re[χT5(3)] are all coincide for different tunneling. With zero energy splitting, the enhanced cross-Kerr nonlinearity occurs at the position of $\delta_{p}=0$ in the vicinity of low linear absorption (Figure 6g). The enhanced cross-Kerr nonlinearity with low linear absorption can also be achieved for nonzero energy splitting, as shown in Figure 6h.

## 4. Conclusions

In conclusion, linear absorption, self-Kerr nonlinearity, fifth-order nonlinearity and cross-Kerr nonlinearity of MQDs controlled by multiple tunneling was investigated. By using the probability amplitude method, general analytic expression of linear and nonlinear susceptibility of the probe field in MQDs was obtained. The multiple tunneling can induce quantum interference among the dressed states and result in multiple TIT windows. In the vicinity of such TIT windows, enhanced self-Kerr nonlinearity, fifth-order nonlinearity and cross-Kerr nonlinearity accompanied by low linear absorption was realized by choosing the tunneling intensity and energy splitting of the exciton states. Realizing enhanced nonlinearity with low absorption in MQDs has essential applications in novel nonlinear optics and quantum information devices.

## Appendix A

The analytical expressions of the first-order, third-order and fifth-order susceptibilities can be obtained by solving Equations (4)–(7). Under the steady-state condition (${\left|a_{0}\right|}^{2}=1$), Equations (4)–(7) goes to:

(A1) $$-\Omega_{p}a_{1}=0,$$

(A2) $$-\Omega_{p}a_{0}-\sum_{n=2}^{N}T_{n}a_{n}+\left(\delta_{p}-i\gamma_{1}\right)a_{1}=0,$$

(A3) $$-T_{n}a_{1}+\left(\delta_{p}-\omega_{1n}-i\gamma_{n}\right)a_{n}=0\;(n=2,3,\ldots ,N),$$

(A4) $${\sum_{n-0}^{N}\left|a_{n}\right|}^{2}=1.$$

From A3:

(A5) $$a_{n}=\frac{T_{n}}{\Gamma_{n}}a_{1}.$$

Substituting Equation (A5) into Equation (A2), then:

(A6) $$a_{1}=\Omega_{p}\frac{1}{\Gamma_{1}-\sum_{n=2}^{N}\frac{T_{n}^{2}}{\Gamma_{n}}}a_{0}.$$

Substituting Equations (A5) and (A6) into Equation (A4), then:

(A7) $${\left|a_{0}\right|}^{2}=\frac{1}{1+\frac{\Omega_{p}^{2}}{{\left|\Gamma_{1}-\sum_{n=2}^{N}\frac{T_{n}^{2}}{\Gamma_{n}}\right|}^{2}}\left(1+\sum_{n=2}^{N}\frac{T_{n}^{2}}{{\left|\Gamma_{n}\right|}^{2}}\right)}.$$

The coherence element between state |0〉 and |1〉 is:

(A8) $$a_{1}a_{0}^{*}=\Omega_{p}\frac{1}{\Gamma_{1}-\sum_{n=2}^{N}\frac{T_{n}^{2}}{\Gamma_{n}}}\cdot {\left|a_{0}\right|}^{2}.$$

Substituting Equation (A7) into Equation (A8), then:

(A9) $$\chi =\frac{a_{1}a_{0}^{*}}{\Omega_{p}}=\frac{1}{\Gamma_{1}-\sum_{n=2}^{N}\frac{T_{n}^{2}}{\Gamma_{n}}}\cdot \frac{1}{1+\frac{\Omega_{p}^{2}}{{\left|\Gamma_{1}-\sum_{n=2}^{N}\frac{T_{n}^{2}}{\Gamma_{n}}\right|}^{2}}\left(1+\sum_{n=2}^{N}\frac{T_{n}^{2}}{{\left|\Gamma_{n}\right|}^{2}}\right)}.$$

Using the Maclaurin formula and expand χ into the fourth-order of $\Omega_{p}$, then:

(A10) $$\chi =\frac{1}{\Gamma_{1}-\sum_{n=2}^{N}\frac{T_{n}^{2}}{\Gamma_{n}}}\cdot \left(1-\frac{\Omega_{p}^{2}}{{\left|\Gamma_{1}-\sum_{n=2}^{N}\frac{T_{n}^{2}}{\Gamma_{n}}\right|}^{2}}\left(1+\sum_{n=2}^{N}\frac{T_{n}^{2}}{{\left|\Gamma_{n}\right|}^{2}}\right)-\frac{\Omega_{p}^{4}}{{\left|\Gamma_{1}-\sum_{n=2}^{N}\frac{T_{n}^{2}}{\Gamma_{n}}\right|}^{4}}{\left(1+\sum_{n=2}^{N}\frac{T_{n}^{2}}{{\left|\Gamma_{n}\right|}^{2}}\right)}^{2}\right).$$

The first-order susceptibilities is proportional to $\Omega_{p}^{0}$, the third-order susceptibilities is proportional to $\Omega_{p}^{2}$, and the third-order susceptibilities is proportional to $\Omega_{p}^{4}$, thus

(A11) $$\chi =\chi^{\left(1\right)}+\chi^{\left(3\right)}\Omega_{p}^{2}+\chi^{\left(5\right)}\Omega_{p}^{4},$$

where

(A12) $$\chi^{\left(1\right)}=-\frac{1}{\Gamma_{1}-\sum_{n=2}^{N}\frac{T_{n}^{2}}{\Gamma_{n}}},$$

(A13) $$\chi^{\left(3\right)}=-\frac{1}{\Gamma_{1}-\sum_{n=2}^{N}\frac{T_{n}^{2}}{\Gamma_{n}}}\cdot \frac{1}{{\left|\Gamma_{1}-\sum_{n=2}^{N}\frac{T_{n}^{2}}{\Gamma_{n}}\right|}^{2}}\cdot \left(1+\sum_{n=2}^{N}\frac{T_{n}^{2}}{{\left|\Gamma_{n}\right|}^{2}}\right),$$

(A14) $$\chi^{\left(5\right)}=-\frac{1}{\Gamma_{1}-\sum_{n=2}^{N}\frac{T_{n}^{2}}{\Gamma_{n}}}\cdot \frac{1}{{\left|\Gamma_{1}-\sum_{n=2}^{N}\frac{T_{n}^{2}}{\Gamma_{n}}\right|}^{4}}\cdot {\left(1+\sum_{n=2}^{N}\frac{T_{n}^{2}}{{\left|\Gamma_{n}\right|}^{2}}\right)}^{2}.$$
